# Basic Characteristics of Flower Transcriptome Data and Derived Novel EST-SSR Markers of *Luculia yunnanensis*, an Endangered Species Endemic to Yunnan, Southwestern China

**DOI:** 10.3390/plants11091204

**Published:** 2022-04-29

**Authors:** Yao Zhang, Xi Liu, Yuying Li, Xiongfang Liu, Hong Ma, Suping Qu, Zhenghong Li

**Affiliations:** 1 Institute of Highland Forest Science, Chinese Academy of Forestry, Kunming 650224, China; zhangyao949@163.com (Y.Z.); liuxiongfang@caf.ac.cn (X.L.); 2 College of Forestry, Nanjing Forestry University, Nanjing 210037, China; 3 School of Geography and Ecotourism, Southwest Forestry University, Kunming 650224, China; lx13320441881@163.com; 4 College of Grassland Science, Shanxi Agricultural University, Jinzhong 030801, China; liyuy_ing@163.com; 5 Key Laboratory of Breeding and Utilization of Resource Insects, National Forestry and Grassland Administration, Kunming 650224, China; 6 Flower Research Institute, Yunnan Academy of Agricultural Sciences, Kunming 650205, China

**Keywords:** *Luculia yunnanensis*, transcriptome, EST-SSR markers, polymorphism, conservation genetics

## Abstract

*Luculia yunnanensis* (Rubiaceae), an evergreen shrub or small tree, is endemic to China and confined to Nujiang Prefecture, Yunnan Province. This plant is of high ornamental value owing to its attractive pink flowers, sweet fragrance, and long flowering period. Due to the influence of climate change and human factors, the distribution range of *L. yunnanensis* has exhibited a significant shrinking trend, and it has become a vulnerable species that is in urgent need of conservation and rational utilization research. In this study, the flower transcriptome sequencing of *L. yunnanensis* was conducted using an Illumina HiSeq platform. We designed and developed a series of EST-SSR primers based on the flower transcriptome data of *L. yunnanensis*. The results showed that 98,389 unigenes were obtained from the *L. yunnanensis* flower transcriptome, all of which were aligned with sequences in public databases. Nr, Nt, Pfam, KOG/COG, Swiss-Prot, KEGG, and GO annotated 31,859, 13,853, 22,684, 10,947, 21,416, 9722, and 23,390 unigenes, respectively. The MISA (Microsatellite) tool was used to identify SSR loci from all unigenes, and a total of 15,384 SSRs were identified. Repeat motifs were given priority with mononucleotides, dinucleotides, and trinucleotides. The 81 primer pairs were synthesized randomly, of which 44 pairs showed effective amplification. A total of 17 primers showed stable amplification, and rich polymorphism was observed in 6 populations. We concluded via genetic diversity analysis that the average effective number of alleles (Ne), Shannon’s information index (I), and polymorphism information content (PIC) were 1.925, 0.837, and 0.403, respectively. In conclusion, 17 EST-SSR primers can be used for subsequent population genetic diversity analysis and molecular-marker-assisted breeding, which is of great significance for formulating resource conservation and utilization strategies for *L. yunnanensis*.

## 1. Introduction

As an endangered species endemic to Yunnan Province, Southwestern China, *Luculia yunnanensis* is an evergreen shrub or small tree that belongs to the genus *Luculia*, of the family Rubiaceae. This species mainly grows on limestone mountains, secondary shrubby woodland, and open slopes at altitudes of between 1200 and 3200 m. It possesses high ornamental value due to its attractive plant shape, long flowering period, striking pink flowers, sweet fragrance, and the fact that it is evergreen. There is thus great value in its development and utilization.

Our previous field investigations of *L. yunnanensis* and its recorded distribution showed an obvious shrinking trend in its distribution range, which currently only comprises Lushui, Fugong, and Gongshan counties in Nujiang Prefecture, Yunnan Province. The distribution range has been reduced from seven counties in five prefectures or cities to three counties in one prefecture, and the administrative area of the distribution area is 10,465.48 km^2^ (only 20% of its historical range). According to the IUCN threat level criteria, *L. yunnanensis* belongs to the vulnerable species category [1]; thus, there is an urgent need for relevant biological conservation research.

The assessment of genetic diversity is one of the most significant parts of the research on the conservation of rare and endangered plants [2]. Considered to be an important component that could be seen as a determination of the viability of a species, it may affect species’ adaptive capacity and evolutionary potential, and is often regarded as an indirect warning of the potential extinction of endangered species [3,4]. A decrease in genetic diversity in a species may lead to the degradation of its adaptability to environmental changes, leading to a decline in its evolutionary ability [5,6]. Thus, only by clarifying the genetic diversity of threatened species and taking various corresponding factors into consideration can we propose feasible strategies regarding their protection and utilization.

DNA molecular markers are usually used to estimate the genetic diversity of plants, and a sufficient number of polymorphic primers is the prerequisite for molecular genetic diversity analysis. As one of the most commonly used molecular marker techniques, EST-SSR has the advantages of simple operation, good stability, high accuracy, rich variability, good repeatability, and interspecific transmissibility [7,8,9,10]. Compared with genomic SSR markers, EST-SSR markers have low development cost and can be applied to species without reference genomes, and the application of EST-SSR markers has greatly promoted the use of molecular marker techniques in plant research [10,11,12], as well as being widely used in fields such as plant genetics and breeding, conservation, and the development of germplasm [13,14,15,16]. To date, the development of EST-SSR primers for *L. yunnanensis* has not been reported. Zhou [17] made use of a modified biotin–streptavidin capture method to develop 13 pairs of SSR primers suitable for analyzing the genetic diversity of *Luculia*. These 13 pairs of primers can effectively distinguish between *Luculia pinceana* and *L. yunnanensis*. With the same method, Ma [18] developed 24 pairs of SSR primers, and 11 pairs of them were polymorphic in two populations of *L. yunnanensis*. However, this number of markers is far from meeting the needs of follow-up studies on *L. yunnanensis*. Therefore, in this study we designed a series of EST-SSR primers based on the flower transcriptome data of *L. yunnanensis*, and developed a set of polymorphic EST-SSR primers that can be used for subsequent population genetic diversity analysis and molecular-marker-assisted breeding, which are of great significance for formulating resource conservation and utilization strategies for *L. yunnanensis*.

## 2. Results and Discussion

### 2.1. Functional Annotation and Classification

The transcriptome sequencing was conducted using an Illumina HiSeq platform (Beijing Novogene Biological Information Technology Co., Ltd., Beijing, China); clean reads were used for de novo assembly using Trinity with the default parameters to obtain 140,042 transcripts, and TGICL version 2.1 software was used to cluster the transcripts into 98,389 unigenes. There were 18,521 unigenes (18.82% of the total unigenes) with a length of more than 1 Kb and high assembly integrity (Table 1). To learn more about the features and functions of these unigenes, we aligned them with the sequences of public databases, including Nr, Nt, Pfam, KOG/COG, Swiss-Prot, KEGG, and GO (see Table 2). It can be seen from the results that at least some of the unigenes were not annotated in all seven databases, which demonstrates that there are unknown unigene sequences. This is consistent with the fact that the annotation of the unigenes of *Glycyrrhiza uralensis* [19], *Luculia gratissima* [20], and *Prunella vulgaris* [21] did not reach 100%. This result may be caused by the length of some unigenes being so short that the annotation information is incomplete, or by the specific unigenes of this species not being fully recognized and the relevant information not being included in the database. The specific reasons for this need to be further studied via sequence analysis and gene expression verification.

**Figure 1:**
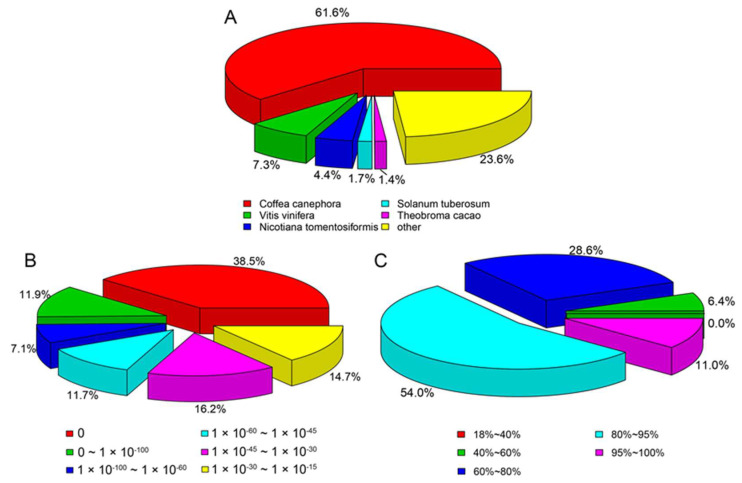
Characterization of assembled *L*. *yunnanensis* unigenes using the Nr database: (**A**) Species distribution for the assembled unigenes. (**B**) E-value distribution for the assembled unigenes. (**C**) Similarity distribution for the assembled unigenes.

According to the species classification from the result of BLAST with Nr (Figure 1A), *Coffea canephora* (Rubiaceae, *Coffea*) had the highest matching rate, followed by *Vitis vinifera* (Vitaceae, *Vitis*), with the next highest being *Nicotiana tomentosiformis* (Solanaceae, *Nicotiana*). As can be seen from Figure 1B, 38.5% of the unigenes can be fully matched to Nr (the smaller the E-value, the higher the degree of matching and the similarity), and the degree of matching in Nr is relatively high. Figure 1C shows that 11.0% of the unigenes had more than 95% similarity, and 54% of the unigenes had 80–95% similarity (the higher the similarity, the higher the confidence).

**Figure 2:**
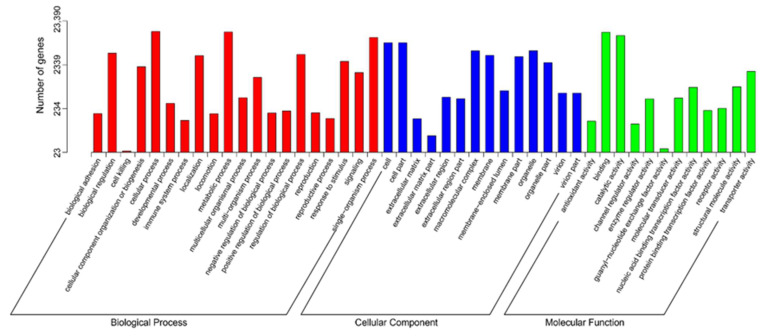
GO classification of assembled unigenes of the *L. yunnanensis* flower transcriptome. The *x*-axis indicates the subgroups in GO annotation; the *y*-axis indicates the number of genes in each category.

**Figure 3:**
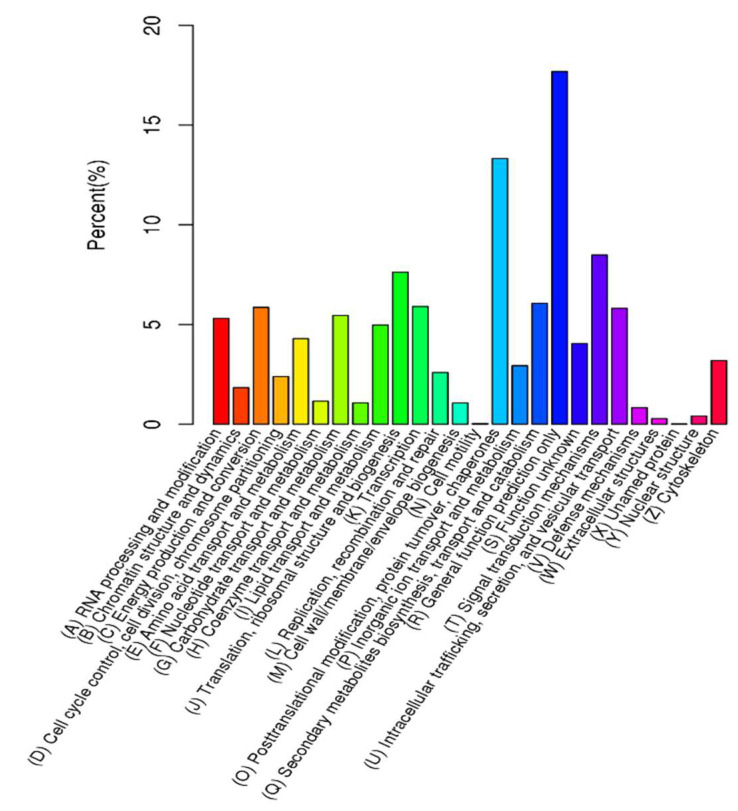
KOG classification of assembled unigenes of the *L. yunnanensis* flower transcriptome. The *x*-axis indicates the 26 groups in KOG annotation; the *y*-axis indicates the percentage of the number of genes in each group relative to the total number of annotated genes.

**Figure 4:**
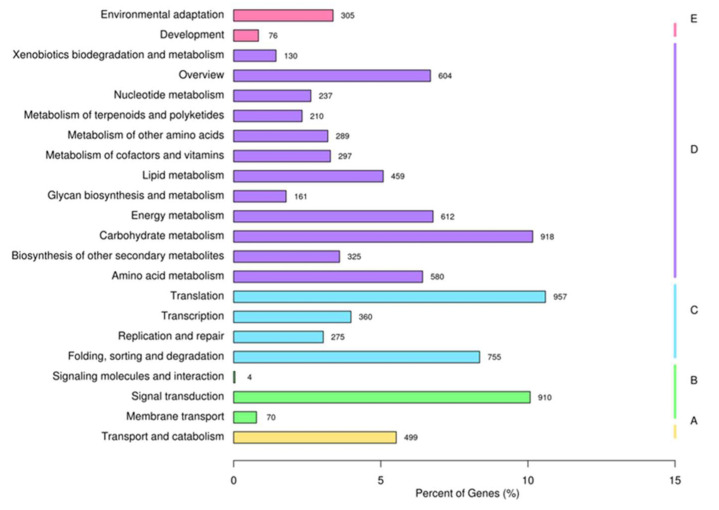
KEGG metabolic pathways of assembled unigenes of the *L. yunnanensis* flower transcriptome: (**A**) cellular processes; (**B**) environmental information processing; (**C**) genetic information processing; (**D**) metabolism; (**E**) organismal systems. The *x*-axis indicates the number of genes in each metabolic pathway and the ratios of the number of genes to total number of annotated genes; the *y*-axis indicates the names of the KEGG metabolic pathways.

All unigenes were assessed for GO assignments based on the Nr annotations. The unigenes were categorized into biological process (BP), cellular component (CC), and molecular function (MF) to reveal the gene function classification (Figure 2). Within these functional groups, “metabolic process” was the dominant group among biological processes (282,588), which contained 144,606 unigenes, and second was “cellular process” (62,514). Among cellular components (83,109), “cell part” (24,619) contained the largest number of unigenes, followed by “organelle” (19,336). With a total of only 57,203 unigenes in the molecular function category, the two largest groups were “binding” and “catalytic activity”, which included 41,251 and 11,311 unigenes, respectively. This result was similar to those for *Lycium barbarum* [22], *Rhododendron fortune* [23], and *Elaeagnus mollis* Diels [24], which showed that metabolic processes and cellular processes contained the largest numbers of unigenes in all subcategories. The genes successfully annotated by KOG were classified into 26 KOG groups, as shown in Figure 3. The dominant group was general function prediction (1936), followed by the posttranslational modification, protein turnover, and chaperones (1459), while the smallest group was cell motility (only 4). The KOG classification results of the three species mentioned above are still consistent with the results of our study. In addition, the group of unknown functions contained 443 unigenes, accounting for 3.6% of the total annotation information in the *L. yunnanensis* flower transcriptome. Studies on *Castanea henryi* (Skan) Rehd. et Wils. [25], *Pinus yunnanensis* Franch. [26], *Rhododendron longipedicellatum* Lei Cai et Y. P. Ma [27], and *Phyllanthus emblica* [28] have produced similar results. We speculate that this may be caused by insufficient annotation information. By comparing the KEGG database, the unigenes can be classified into 22 KEGG pathways according to the signaling pathways involved. Of these pathways, the most represented was translation (957), followed by carbohydrate metabolism (918) and signal transduction (910) (Figure 4).

The unigenes were roughly divided into 3 functional categories and 56 subcategories according to GO function, among which metabolic process, cellular process, cell, cell part, organelle, binding, and catalytic activity were highly enriched. In other words, the expression levels of genes related to cellular activity, metabolic activity, and catalytic activity was high, indicating that *L. yunnanensis* has strong metabolic capacity. We hypothesized that this may be related to the continuous cell proliferation of the meristem during flower development and the vigorous metabolic activities in the flower organs of *L. yunnanensis*. Xia et al. [29] analyzed the flower transcriptome of *Camellia sinensis*, and their results and hypotheses were similar to ours. KEGG functional annotation analysis showed that unigenes could be grouped into 5 categories, among which the pathways related to metabolism and genetic information processing accounted for the highest proportion, and the number of genes related to metabolism-related pathways was the largest. This was further proof that there was strong metabolic activity in *L. yunnanensis* during this period. Additionally, the metabolic pathways of amino acid metabolism, carbohydrate metabolism, lipid metabolism, biosynthesis of other secondary metabolites, and environmental adaptation were involved in the KEGG functional annotation analysis. These data provide a molecular basis for further research on resistance mechanisms, and allow us to explore the genes related to the flowering regulation and environmental adaptability of *L.* *yunnanensis*.

### 2.2. Identification of SSRs

The MISA (Microsatellite) tool was used to identify SSR loci in the transcriptome data, and a total of 15,384 SSRs were identified from 98,389 unigenes; the SSR frequency in the transcriptome was 15.63%, and the mean distance of the SSRs in the unigenes was 6.39 kb. It is well known that the distribution frequency of SSR loci varies greatly between different species, which may be related to the genome size of species, the organ and period of the sampled plant used for transcriptome sequencing, the SSR development methods, and the screening criteria [30]. Species with higher frequencies include, for instance, hybrid *Cymbidium* (58.64%) [31], *Morus alba* L. (45.56%) [32], and *Bougainvillea glabra* (44.91%) [33]. Species with lower frequencies include *Paeonia suffruticosa* (6.4%) [34], *Pinus elliottii* (4.80%) [35], and *Chrysanthemum morifolium* (2.84%) [36]. Kumpatal [37] found that the distribution frequency of SSR loci in dicotyledons ranged from 2.65% to 16.82%, with an average of 9.73%. The distribution frequency of SSR loci in the flower transcriptome of *L. yunnanensis* is consistent with the distribution frequency range of SSR loci in dicotyledons, and higher than the average. Therefore, the flower transcriptome data of *L. yunnanensis* had abundant SSR loci, and could be used for the development of SSR markers.

There were abundant SSR types in the flower transcriptome of *L. yunnanensis*, with the distribution of mononucleotide to hexanucleotide repeat types, but the proportion of different repeat types was significantly different. The number of mononucleotide repeat motifs found to be the most frequent was 8889, accounting for 60.29% of the total repeat motifs. Second, dinucleotide and trinucleotide repeat motifs were 3340 and 2309, accounting for 22.65% and 15.66%, respectively, of the total repeat motifs. The tetranucleotide, pentanucleotide, and hexanucleotide repeat motifs accounted for only 158, 23, and 25, respectively. In this study, the number of short-repeat motifs (mononucleotide, dinucleotide, and trinucleotide) was more than that of long-repeat motifs, which is consistent with the distribution in most species, and supports the view that long-repeat motifs have high variability [38]. As shown in Table 3, the mono-, di-, tri-, tetra-, penta-, and hexanucleotide motifs with the highest frequency were A/T, AG/CT, AAG/CTT, AAAG/CTTT, CCTTC/GAAGG, and GAGAC/GTCTC, respectively. The frequency of mononucleotide repeats was the highest. This result was similar to those for *Ricinus communis* [39], *Glycyrrhiza uralensis* Fisch [40], *Arachis hypogaea* L. [41], and *Elaeagnus mollis* Diels [24], which showed that the A/T repeat motif accounted for the largest proportion of SSRs. The proportion of 12–21 bp repeats in SSRs under different repeat types and repeat times was more than 95%, among which the proportion of 5 times and trinucleotide repeat motifs was the largest (22.25%), followed by that of 6 times and dinucleotide repeat motifs (16.48%) (Table 4).

The polymorphism level of SSR markers is an important basis for evaluating their availability, and the length of SSRs is an important factor affecting their polymorphism. The results showed that the polymorphism was high when the length of SSRs was more than or equal to 20 bp, medium when the length was 12–19 bp, and extremely low when the length was less than 12 bp [42]. Therefore, more than 95% SSRs were had a moderate-to-high level of polymorphism in this study. In addition, lower-grade SSR motifs (i.e., mononucleotide, dinucleotide, and trinucleotide) tend to produce polymorphism more easily than higher-order SSR motifs [43,44]. Due to the large number of mononucleotides, dinucleotides, and trinucleotides in this study, it can be predicted that SSRs obtained from the *L. yunnanensis* flower transcriptome have a high potential for polymorphism, and this leads to a high application value in the study of its molecular markers.

### 2.3. SSR Primer Screening and Verification

**Figure 5:**
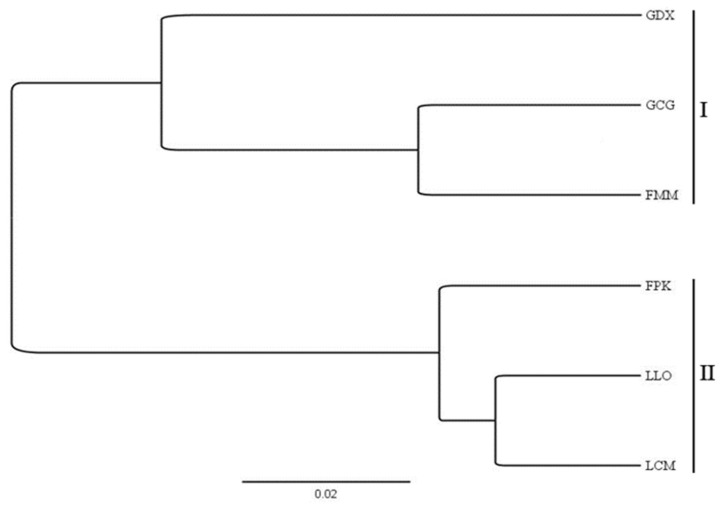
Cluster analysis of 6 *L. yunnanensis* populations based on 17 EST-SSR markers.

A total of 9195 primer pairs were designed using Primer 3.0, and 81 primer pairs were randomly selected to amplify across the genomic DNA template of *L. yunnanensis*. Of the 81 primer pairs, 44 of them successfully amplified fragments from *L. yunnanensis* genomic DNA, with a success rate of 54.32%. Therefore, the primers developed and designed in this study have a certain applicability to the population of *L. yunnanensis*. According to the preliminary screening results, 10 individuals were randomly selected from each population (a total of 60 individuals from 6 populations) to further verify the polymorphism of the 44 primer pairs that could amplify bands. Among them, 17 primer pairs were found to be polymorphic in *L. yunnanensis*, producing clear amplicons of the expected size, which were suitable for the detection of SSR loci by capillary electrophoresis. The specific information of 17 EST-SSR primers is shown in Table 5. A dendrogram clustered the 6 populations of *L. yunnanensis* into 2 clusters (Figure 5). One cluster (I) included 2 populations (GDX and GCG) of Gongshan County and one population (FMM) of Fugong County. The other cluster (II) included 2 populations (LCM and LLO) of Lushui County and one population (FPK) of Fugong County. Geographically, FMM is close to Gongshan County and FPK is close to Lushui County, which is consistent with the results of cluster analysis.

The SSR marker diversity index was estimated using POPGENE 1.32; the effective number of alleles (Ne) and Shannon’s information index (I) per locus varied from 1.298 to 2.690 (average of 1.925), and from 0.476 to 1.239 (average of 0.837), respectively. Using CERVUS 3.0 (Field Genetics, London, UK), we calculated that the allelic polymorphism information content (PIC) for each SSR locus ranged from 0.223 to 0.580, with an average of 0.403. Botstein [45] put forward a criterion that primers are highly polymorphic when PIC > 0.5, moderately polymorphic when 0.5 > PIC > 0.25, and low-grade polymorphic when PIC < 0.25. Most of the 17 EST-SSR primer pairs were moderately or highly polymorphic, indicating that these primers could be used for genetic diversity analysis and fingerprint construction of *L. yunnanensis*.

Today, plant diversity is gradually decreasing on a global scale, leading to a related decline in ecosystem service function [46]. Many rare species, such as *L. yunnanensis*, have seldom been studied, and lack protection due to a lack of social awareness. Thus, the numbers and distribution ranges of the species are shrinking. Using transcriptome sequencing technology to study the genetic diversity of *L. yunnanensis* can quickly and efficiently develop mass EST-SSR markers. The application of these markers is conducive to quickly supplementing the molecular biological information of this species, and provides a theoretical basis and scientific evidence for research on the genetic resources, conservation, and utilization of *L. yunnanensis*. In addition, due to the high ornamental value of *L. yunnanensis*, we can refer to Ding’s [47] study on *Chrysanthemum*, in which the development of SSR markers for genes related to the regulation of flower shape and color were studied, and these SSR markers were applied to the classification of more than 100 *Chrysanthemum* germplasm resources with different flower colors and shapes. In the same way, we could also screen the SSR loci of important ornamental traits such as the flower shape and color of *L. yunnanensis*, which could lay the foundation for breeding new varieties of *L. yunnanensis*.

## 3. Materials and Methods

### 3.1. Plant Materials

We picked the experimental materials from Nujiang Lisu Autonomous Prefecture, Yunnan Province. Inflorescences of *L. yunnanensis* were collected, bottled, and brought back to Kunming for subsequent use. The blooming flowers of five individuals collected within LLO (Table 6) were frozen immediately in liquid nitrogen and stored at −80 °C for use as transcriptome sequencing materials. The plant samples were mixed, and RNA was extracted using an RNA extraction kit from TransGen (Beijing, China). Transcriptomic sequencing was performed at Beijing Novogene Biological Information Technology Co., Ltd., Beijing, China. Based on the results of previous investigations into the resource status of *L. yunnanensis*, 6 populations were selected as primer polymorphism detection materials in regions with a dense distribution and adequate quantity, and where sampling was convenient and relatively representative. These were GDX, GCG, FMM, FPK, LLO, and LCM (Table 6).

### 3.2. De Novo Assembly, Functional Annotation, and Classification

Transcriptome sequencing was carried using an Illumina HiSeq 4000 platform (Beijing Novogene Biological Information Technology Co., Ltd., Beijing, China); clean reads were used for de novo assembly using Trinity (version: trinityrnaseq-r20131110) with the default parameters to obtain transcripts, and TGICL version 2.1 software was used to cluster the transcripts into unigenes [48,49]. For functional prediction and classification, all unigenes were compared against public databases, including NCBI non-redundant protein sequences (Nr), Nt, Protein Family (Pfam), Eukaryotic Orthologous Groups of Proteins (KOG/COG), Swiss-Prot, the KEGG Orthology database (KO), and Gene Ontology (GO). BLAST [50] was used for unigene annotation in the Nt, Nr, COG, and Swiss-Prot databases. The KEGG Automatic Annotation Server [51] was used for KEGG annotation of unigenes. Blast2GO [52] and Nr annotation results were used for the GO annotation.

### 3.3. Detection of EST-SSR Markers and Designing of Primers

SSR loci in the transcriptome were identified using the microsatellite identification tool MISA (http://misaweb.ipk-gatersleben.de/) (accessed on 3 December 2020) [53]. The parameters of identifying the mono-, di-, tri-, tetra-, penta-, and hexanucleotide motifs were set to minimum numbers of 10, 6, 5, 5, 5, and 5 repeat units, respectively. Primer pairs were designed using Primer 3.0 software [54], according to the following parameters: the length range of the optimal primer sizes was 18–23 bp, the optimal annealing temperature was from 55–65 °C, the PCR product size range was 80–300 bp, and the GC content was 40–60%.

### 3.4. DNA Extraction and Quality Check

Total genomic DNA was extracted from the silica-dried leaves of *L. yunnanensis* with a plant genomic DNA extraction kit containing RNase A (TransGen, Beijing, China), following the manufacturer’s protocol. The integrity and quality of the genomic DNA was checked using 0.8% agarose gel electrophoresis. The DNA purity and concentration were determined using a NanoDrop 2000 spectrophotometer (Thermo Fisher Scientific, Wilmington, DE, USA), and samples were stored at −20 °C until used for SSR amplification.

### 3.5. EST-SSR Amplification and Data Analysis

The 81 pairs of randomly selected SSR primers were used for preliminary screening, and 10 individuals per population were used to test the primers amplified with clear bands in order to further determine the polymorphism of the SSR primers. PCR amplification was carried out in a 10 μL reaction mixture containing 10–30 ng of genomic DNA, 0.15 mmol/L of each dNTP, 1 μL of 10×Taq buffer, 0.6 mmol/L each of reverse and forward primer, and 1 unit of Taq DNA polymerase (TransGen, Beijing, China). The PCR amplification was carried out using a Bio-Rad C1000 Touch ™ Thermal Cycler (Bio-Rad Laboratories, CA, USA), as follows: 5 min of initial denaturation at 94 °C, followed by 30 cycles of denaturation for 30 s at 94 °C, a temperature gradient for annealing from 50 °C to 60 °C for 30 s, and extension at 72 °C for 1 min, with a final 10 min at 72 °C for final extension. The PCR products were resolved by electrophoresis in 1.5% agarose gels to determine whether amplification was successful. PCR fluorescent tagging and capillary electrophoresis were performed to further screen polymorphisms. The 5′ end of each forward primer was labeled with FAM or HEX fluorescent dyes (Thermo Fisher Scientific, Wilmington, DE, USA). The PCR amplifications were performed using the PCR conditions mentioned above. The multiplex DNA products labeled with the above-mentioned fluorescent dyes were analyzed on an ABI 3730xl DNA Analyzer with a GeneScan 500 LIZ Size Standard (Thermo Fisher Scientific, Wilmington, DE, USA), and allele sizes were assessed using GeneMapper 4.1 (Thermo Fisher Scientific, Wilmington, DE, USA). The parameters of genetic diversity—including the number of alleles (Na), effective number of alleles (Ne), and Shannon’s information index (I)—of these SSR loci were estimated using POPGENE 1.32 [55], and the allelic polymorphism information content (PIC) for each SSR locus was calculated using CERVUS 3.0 (Field Genetics, London, UK) [56]. Cluster analysis of 6 populations on the basis of shared allele distance (DAS) was performed using POPULATIONS 1.2.30 [57], with the unweighted pair group method with arithmetic mean (UPGMA) method. The clustering tree was visualized and edited using Interactive Tree of Life (iTOL) version 3 [58]. The primers with good repeatability, clear bands, and high polymorphism were selected as candidate primers for molecular genetic diversity research on *L. yunnanensis*.

## 4. Conclusions

The *L. yunnanensis* transcriptome was obtained using an Illumina HiSeq platform. In total, 98,389 unigenes were generated, of which 98,389 unigenes were aligned with the sequences of public databases. Nr, Nt, Pfam, KOG/COG, Swiss-Prot, KEGG, and GO annotated 31,859, 13,853, 22,684, 10,947, 21,416, 9722, and 23,390 unigenes, respectively. EST-SSR specific primer pairs were successfully designed. In addition, MISA was used to identify SSR loci from all unigenes, and a total of 15,384 SSRs was identified. The repeat motif was given priority with mononucleotides, dinucleotides, and trinucleotides. Furthermore, the 81 primer pairs were synthesized randomly, of which 44 pairs showed effective amplification, 17 primers showed stable amplification, and rich polymorphism was observed in 6 populations. This sequence resource and these potential EST-SSR markers can be used for subsequent population genetic diversity analysis and marker-assisted breeding, which is of great significance for formulating resource conservation and utilization strategies for *L. yunnanensis*.

## Figures and Tables

**Table 1:** Length distribution after assembly in the flower transcriptome of *L. yunnanensis*.

Transcript Length Interval	200–500 bp	500–1 kbp	1 k–2 kbp	>2 kbp	Total
Number of transcripts	71,181 (50.83%)	25,812 (18.43%)	23,641 (16.88%)	19,408 (13.86%)	140,042
Number of unigenes	62,121 (63.14%)	17,747 (18.04%)	10,870 (11.05%)	7651 (7.78%)	98,389

**Table 2:** The statistics of the unigenes’ annotation in the flower transcriptome of *L. yunnanensis*.

Annotation	Number of Annotated Unigenes	Percentage of Annotated Unigenes (%)	Percentage of Total Unigenes (%)
Annotated in Nr	31,859	32.38	22.75
Annotated in Nt	13,853	14.07	9.89
Annotated in KO	9722	9.88	6.94
Annotated in Swiss-Prot	21,416	21.76	15.29
Annotated in Pfam	22,684	23.05	16.20
Annotated in GO	23,390	23.77	16.70
Annotated in KOG	10,947	11.12	7.82
Annotated in all databases	4273	4.34	3.05
Annotated in at least one database	36,497	37.09	26.06
Total annotated unigenes	98,389	100	70.26

**Table 3:** Frequency of different repeat motifs in the SSRs of the *L. yunnanensis* flower transcriptome.

Repeat Types	No.	Frequency (%)	Maximum Repeat Motif (Number and %)
Mononucleotide repeat (p1)	8889	60.29	A/T (8816, 99.18%)
Dinucleotide repeat (p2)	3340	22.65	AG/CT (2174, 65.09%)
Trinucleotide repeat (p3)	2309	15.66	AAG/CTT (428, 18.54%)
Tetranucleotide repeat (p4)	158	1.07	AAAG/CTTT (22, 13.92%)
Pentanucleotide repeat (p5)	23	0.16	CCTTC/GAAGG (2, 8.70%) GAGAC/GTCTC (2, 8.70%)
Hexanucleotide repeat (p6)	25	0.16	CCTTC/GAAGG (2, 8.70%) GAGAC/GTCTC (2, 8.70%)

**Table 4:** Number of SSRs of different repeat motifs on different repeat times in the flower transcriptome of *L. yunnanensis*.

Repeat Types	Repeat Times						
	5	6	7	8	9	10	>10
Dinucleotide repeat	—	965	688	558	591	417	121
Trinucleotide repeat	1303	604	374	23	5	—	
Tetranucleotide repeat	132	25	—	—	—	1	—
Pentanucleotide repeat	14	6	2	—	1	—	—
Hexanucleotide repeat	7	9	5	4	—	—	—

**Table 5:** The information of 17 EST-SSR primers of *L. yunnanensis*.

Primer ID	Primer Sequence (5′-3′)	Repeat Motif	*Ta* (°C)	Size (bp)	Na	Ne	*I*	PIC
N3	CAAATTCGCGCACCAAAACG	(TGGCGT)5	52	261–279	4	2.538	1.061	0.542
	GCTAGAGAGAAAAGGGGCCG							
N6	ACTGCGTACCTCTCCCTCTT	(TAATT)5	58	212–228	5	1.933	0.890	0.426
	TCTCTCTCTCTCGGACGGAC							
N9	GACCCCAAGTTGGCTGATCA	(TGTTAC)8	60	127–187	6	2.212	0.923	0.446
	AGGGCACTTCTGTCATTTCGA							
N10	CTGGTGCACGAGGATTGAGT	(TCAATT)7	60	194–224	2	1.427	0.476	0.255
	GAAGAGTGCCATGGAAACTGC							
Z13	CCTCCCATAGCAGCAGCAAT	(ACC)5	54	117–132	5	1.699	0.833	0.382
	AGTAGTATTAATAATGGCTGGAGGT							
N22	CGCTTCTGTGTTCGAAACCA	(ACAAC)7	60	165–180	4	2.575	1.091	0.549
	CAAAGCTTCCCGTCAACAGC							
N24	CCCACCGAGCAATACCCAAA	(GAAA)5	56	268–280	4	1.344	0.555	0.245
	ACCTTCTCTGTACTCTGCCT							
Z31	GCAATCCTACTCGTGCTGGT	(GGC)6	54	228–240	5	2.583	1.239	0.580
	AGCCAAGACTCGGCAGAAAA							
Z32	TGCACTCCATAAAAGAAGAAAACACA	(TATT)5	53	114–122	3	1.405	0.544	0.264
	TGCAGTAACTTCGTGCCCTT							
Z33	CCCAACCCACCACACAAGT	(TCT)6	54	255–270	5	1.749	0.887	0.404
	AGAGAGGAGGATCGAGGACG							
Z36	TCGGGTCCTAGGGCTTTCTT	(CTTT)5	54	211–219	3	1.766	0.768	0.390
	GGCCCTCCTTGAGCATTGAT							
Z38	ACCCAAGGAACTCTGTCTCT	(AAT)6	53	109–118	4	1.522	0.684	0.321
	ACACTTTCGTCGTCCTTAGGT							
N41	GCCAGAAGGATAGCTTTCGC	(TCCT)5	53	197–221	6	1.298	0.547	0.223
	GGTTTGTGGTGGTTTTTGGGA							
Z48	AGGAAGGGCTTGTTTTTAAGGT	(AG)8	52	215–225	6	1.724	0.914	0.400
	GAGCCAATGACGATCCAGCT							
Z50	TCTGCTGCATCCAATGTACTGT	(GT)8	54	144–154	5	2.690	1.205	0.576
	CCTGCCATAGGTGCCCATTT							
Z54	AGTAAGTGGGTGGAGGTGGT	(TTGA)5	59	212–216	2	1.910	0.670	0.363
	AGGGGCTGATTCTCTAGCGA							
X70	AGCTGGAAACTAAAGGTGGAGG	(ATC)5	58.5	244–250	3	2.355	0.944	0.493
	CTCAGTCTGTCAGGCCTGTG							

Na = number of different alleles; Ne = number of effective alleles; *I* = Shannon’s information index; PIC = polymorphism information content; *Ta* = annealing temperature.

**Table 6:** The location and habitat of the 6 *L. yunnanensis* populations.

Code	GDX	GCG	FMM	FPK	LLO	LCM
Location	Dulongjiang township, Gongshan County	Cikai town, Gongshan County	Maji township, Fugong County	Pihe township, Fugong County	Luobenzhuo township, Lushui County	Chenggan township, Lushui County
Altitude (m)	1324–1788	1325–1778	1354–1455	1738–1752	2029–2153	1664–1736
Longitude and latitude (°)	98.3188 −98.3466 /27.6915 −27.9160	98.6088 −98.8334 /27.5254 −27.7472	98.8388 −98.8453 /27.3632 −27.3651	98.9102 −98.9106 /26.4799 −27.4801	98.8116 −98.8172 /26.4780 −26.4812	98.8253 −98.8312 /26.3540 −26.3563

## Data Availability

Not applicable.
